# Synthesis of Novel Lipophilic *N*-Substituted Norcantharimide Derivatives and Evaluation of Their Anticancer Activities

**DOI:** 10.3390/molecules19066911

**Published:** 2014-05-26

**Authors:** Jin-Yi Wu, Cheng-Deng Kuo, Chien-Yu Chu, Min-Shin Chen, Jia-Hua Lin, Yu-Jen Chen, Hui-Fen Liao

**Affiliations:** 1 Department of Microbiology, Immunology and Biopharmaceutics, College of Life Sciences, National Chiayi University, Chiayi 60004, Taiwan; 2 Laboratory of Biophysics, Department of Medical Research, Taipei Veterans General Hospital, Taipei 11217, Taiwan; 3 Department of Radiation Oncology, Mackay Memorial Hospital, New Taipei City 25160, Taiwan; 4 Institute of Transitional Medicine, National Yang Ming University, Taipei 11221, Taiwan; 5 Department of Biochemical Science and Technology, College of Life Sciences, National Chiayi University, Chiayi 60004, Taiwan

**Keywords:** norcantharimide derivatives, lipophilic substitution, terpenyl group, anticancer activity, HepG2, apoptosis

## Abstract

This research attempted to study the effect of lipophilicity on the anticancer activity of *N*-substituted norcantharimide derivatives. Twenty-three compounds were synthesized and their cytotoxicities against five human cancer cell lines studied. The lipophilicity of each derivative was altered by its substituent, an alkyl, alkyloxy, terpenyl or terpenyloxy group at the *N*-position of norcantharimide. Further, among all synthesized derivatives studied, the compounds *N*-farnesyloxy-7-oxabicyclo[2.2.1]heptane-2,3-dicarboximide (**9**), and *N*-farnesyl-7-oxabicyclo[2.2.1]heptane-2,3-dicarboximide (**18**), have shown the highest cytotoxicity, anti-proliferative and apoptotic effect against human liver carcinoma HepG2 cell lines, yet displayed no significant cytotoxic effect on normal murine embryonic liver BNL CL.2 cells. Their overall performance led us to believe that these two compounds might be potential candidates for anticancer drugs development.

## 1. Introduction

**Figure 1:**
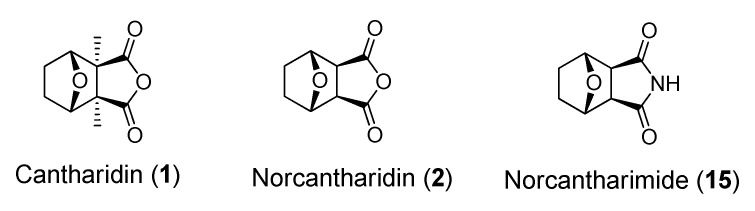
Chemical structures of cantharidin (**1**), norcantharidin (**2**) and norcantharimide (**15**).

Mylabris (*Mylabris phalerata* and *M. cichorii*), the dried body of the blister beetle, has been used in Chinese medicine for thousands of years for the treatment of malignant tumors such as hepatoma, breast cancer, colorectal cancer and abdominal malignancy (Figure 1) [1,2,3]. 

Cantharidin (*exo*-2,3-dimethyl-7-oxabicyclo[2.2.1] heptane-2,3-dicarboxylic acid anhydride), one of the active compounds obtained from Mylabris, has been shown to have anticancer properties both *in vitro* and *in vivo* [4,5,6,7,8,9,10]. It is a potent serine/threonine protein phosphatase 1 (PP1) and protein phosphates 2A (PP2A) inhibitor [11,12,13,14]. Nevertheless, cantharidin (**1**) was also found to be poisonous to the kidney and liver [15]. Though norcantharidin (**2**), the demethylated form of cantharidin, appears to have less nephrotoxicity and liver toxicity, the demethylation also lowers its bioactivity. Meanwhile, studies had also shown that norcantharidin has anticancer activity against various cancer cell lines through the retardation of cell cycle progression and the inhibition of cell proliferation *in vitro*. Further, even though it induces apoptosis in various human cancer cell lines, including melanoma, cervical cancer, bladder cancer, leukemia, colon cancer, breast cancer, and hepatoma [16,17,18,19,20,21,22,23,24,25,26,27,28], the main use of norcantharidin has been limited to the treatment of hepatoma [29,30,31,32]. Lastly, to enhance its anticancer activity, various structural modifications of norcantharidin had been studied and mixed results had been reported. For instance, an analog of norcantharidin was shown to inhibit the function of protein phosphatase and had anti-proliferative activity while others bioactivities were erased [33,34,35,36,37,38,39]. In the previous study, it has been reported that norcantharimide analogues bearing a long alkyl chain at *N*-position may have enhanced bioavailability and transportability through cell membrane. The highly hydrophobic nature of the alkyl tails of norcantharimide analogues may improve their uptake and biological activity [40]. In this study, we examined closely the effect of the lipophilicity of *N*-substituted norcantharimide on their anticancer activities. Specifically, we prepared and evaluated the cytotoxicity of a series of *N*-substituted norcantharimide derivatives that bear alkyl, alkyloxy, terpenyl or terpenyloxy groups at the *N*-position of norcantharimide.

## 2. Results and Discussion

### 2.1. Chemistry

**Scheme 1:**
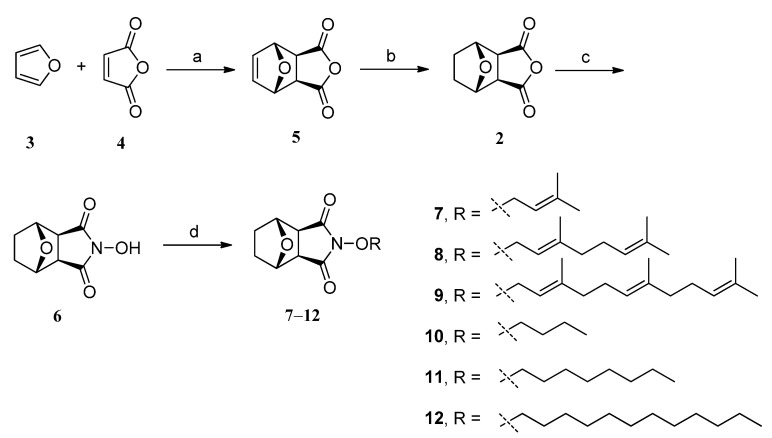
Synthesis of norcantharidin (**2**) and compounds **5**–**12**.

The key starting material in this study was 5,6-dehydronorcantharidin (**5**), which was prepared on a large scale through *exo*-selective cycloaddition, or Diels-Alder reaction, of furan (**3**) and maleic anhydride (**4**). Subsequent hydrogenation of **5** (H_2_, 10% Pd/C) using a modified procedure of McChuskey *et al.* [33] provided the starting norcantharidin (**2**) in excellent yield, which then was employed as the substrate for the synthesis our designed derivatives. First, norcantharidin (**2**) was reacted with hydroxylamine hydrochloride in the presence of sodium methoxide in dry methanol at room temperature to produce *N*-hydroxynorcantharimide (**6**), according to a previous report. *N*-Hydroxynorcantharimide (**6**) was then reacted with terpenyl or alkyl bromide in dry acetone in the presence of K_2_CO_3_ to afford *NO*-substituted derivatives **7**–**12** in moderate to good yields, as shown in Scheme 1.

*Reagents and conditions*: (a) Ether, room temperature, 48 h; (b) 3 atm H_2_, 10% Pd/C, THF, room temperature, 8 h; (c) NaOCH_3_, NH_2_OH∙HCl, MeOH, room temperature, 20 h; (d) K_2_CO_3_, alkyl or terpenyl bromide, acetone, reflux, 8–10 h.

**Scheme 2:**
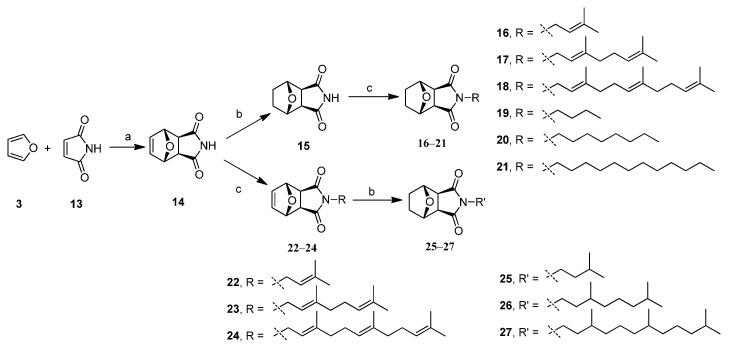
Synthesis of compounds **14**–**27**.

Second, furan (**3**) and maleimide (**13**) were heated in toluene to afford dihydroxynorcantharimide (**14**) by the Diels-Alder reaction. Hydrogenation was then performed as a reduction in dry THF in the presence of catalytic 10% Pd/C to give norcantharimide (**15**). The lipophilic substitution of these derivatives all took place at *N*-position of norcantharimide. The compounds **6**, **14** or **15** reacted with alkyl or terpenyl bromide in dry acetone in the presence of K_2_CO_3_ to produce *N*-substituted derivatives **16**–**24** in moderate to excellent overall yields, as shown in Scheme 2. Using compounds **22**–**24** as precursors we obtained compounds **25**–**27** in moderate to excellent yields through hydrogenation reactions. The synthetic reactions were also outlined in Scheme 2. The chemical structures of these compounds were elucidated by ^1^H-NMR, ^13^C-NMR, 2D NMR and LC-MS spectroscopic methods.

*Reagents and conditions*: (a) Toluene, 80 °C, 6 h; (b) 3 atm H_2_, 10% Pd/C, THF, room temperature, 8–48 h; (c) K_2_CO_3_, alkyl or terpenyl bromide, acetone, reflux, 8–10 h.

### 2.2. In Vitro Anti-Proliferative Activity by MTT Assay

The cytotoxic effects of the twenty-three norcantharimide derivatives, with 5-fluorouracil (5-Fu), cisplatin and doxorubicin as positive control, against five human cancer cell lines was evaluated by the 3-[4,5-dimethylthiazol-2-yl]-2,5-diphenyltetrazolium bromide (MTT) assay [41]. The cancer cells under study were HepG2 (liver carcinoma), BFTC905 (bladder carcinoma), HT29 (colon carcinoma), SW480 (colon carcinoma), and HL60 (leukemia). The data were summarized in Table 1. Of the twenty-three compounds tested, we found eleven compounds; **7**–**9**, **17**, **18**, **20**, **21**, **23**, **24**, **26**, and **27**, **that** showed significant cell growth inhibition on all five human cancer cells. As a trend, the anticancer activity of these compounds increased with the chain length of the substitutes which implied that the lipophilicity of the norcantharimide derivatives at *N*-substitute affected the bioactivity of the compounds. This result indicated that the lipophilic characteristics at *N*-position substitution of the terpenyl moieties of norcantharimide derivatives can remarkably enhance its anticancer activity on a panel of human cancer cell lines. Interestingly, a higher calculated lipophilicity values (clog*P*) of norcantharimide derivatives is associated with a higher anticancer activity, which the longer terpenyl moieties may enhance their bioavailability and improve their cell membrane permeability to raise their cytotoxic activities. In particular, among the eleven bioactive ones, compounds **9**, *N*-farnesyloxy-7-oxabicyclo[2.2.1]heptane-2,3-dicarboximide, and **18**, *N*-farnesyl-7-oxabicyclo[2.2.1]heptane-2,3-dicarboximide, exhibited the highest activities against human liver carcinoma HepG2 cell lines, with the IC_50_ values of 8.3 ± 1.3 and 16.4 ± 1.2 μM, respectively. Compared to norcantharidin (**2**)’s IC_50_ value of 42.0 ± 1.8 μM, these two derivatives evinced a two to five fold stronger cytotoxicity potency against human liver carcinoma HepG2 cells. Meanwhile, when treated with norcantharidin (**2**), compounds **9** or **18**, no significant cell death was detected in normal murine embryonic liver BNL CL.2 cell lines. Furthermore, only a marked effect on cell death (under 20%) was observed at the maximum concentration (60 μM) of these compounds after 24 h or 48 h treatment (Figure S1), except for norcantharidin (**2**) which exhibited slight toxic effect after 48 h. It thus appeared that both compounds **9** and **18** are cytotoxic to human liver cancer cells with no significant adverse effects on normal murine embryonic liver cells. In other words, through a simple structure-activity relationship (SAR) analysis we demonstrated that the cell growth inhibitory potency closely related to the length of the alkyl or terpenyl chain.

**Table 1:** Cytotoxic effects (IC50 values in μM) of norcantharidin (**2**) and its derivatives (**5**–**12**, **14**–**27**), 5-Fu, cisplatin and doxorubicin against five human cancer cell lines for 48 h.

Compound	clog*P* ^a^	IC50 (μM) (Mean ± SD)				
		HepG2 ^b^	BFTC905 ^c^	HT-29 ^d^	SW480 ^d^	HL-60 ^e^
NCTD (**2**)	−0.86	42.0 ± 1.8	18.9 ± 0.3	19.5 ± 0.2	49.1 ± 8.4	ND ^f^
**5**	−1.14	ND	75.3 ± 2.4	ND	ND	ND
**6**	−1.64	ND	ND	ND	ND	ND
**7**	1.23	83.2 ± 2.2	31.7 ± 2.6	38.5 ± 5.9	66.3 ± 2.4	ND
**8**	3.26	32.5 ± 1.4	24.7 ± 1.8	22.0 ± 0.1	44.7 ± 2.3	90.2 ± 2.2
**9**	5.29	8.3 ± 1.3	11.3 ± 1.0	9.7 ± 1.7	18.5 ± 2.3	39.0 ± 1.1
**10**	1.11	ND	ND	ND	ND	ND
**11**	3.23	ND	ND	ND	ND	ND
**12**	5.35	ND	ND	ND	ND	ND
**14**	−1.35	ND	ND	ND	ND	ND
**15**	−1.67	ND	ND	ND	ND	ND
**16**	1.05	ND	ND	ND	ND	ND
**17**	3.09	34.3 ± 4.9	15.5 ± 3.9	26.9 ± 2.2	86.6 ± 2.6	ND
**18**	5.12	16.4 ± 1.2	9.3 ± 0.6	14.8 ± 1.9	33.1 ± 1.0	79.8 ± 1.1
**19**	0.94	ND	ND	ND	ND	ND
**20**	3.06	67.2 ± 8.5	25.8 ± 1.2	57.4 ± 1.6	84.6 ± 1.3	ND
**21**	5.17	34.0 ± 2.7	21.9 ± 0.7	21.9 ± 0.9	46.8 ± 3.0	81.0 ± 2.1
**22**	0.77	ND	ND	ND	ND	ND
**23**	2.80	39.1 ± 2.5	53.0 ± 3.4	54.9 ± 1.5	ND	ND
**24**	4.83	16.5 ± 2.5	10.2 ± 2.8	20.2 ± 3.3	42.8 ± 0.4	ND
**25**	1.34	ND	ND	ND	ND	ND
**26**	3.85	36.4 ± 1.5	ND	28.6 ± 2.1	ND	ND
**27**	6.37	28.1 ± 8.3	13.5 ± 1.9	27.8 ± 4.5	33.8 ± 1.9	ND
5-Fu	−1.72	40.2 ± 7.6	ND	ND	32.7 ± 8.3	ND
Cisplatin	−2.50	36.1 ± 3.1	- ^g^	24.1 ± 0.1	40.7 ± 1.2	ND
Doxorubicin	0.87	0.3 ± 0.0	- ^g^	1.7 ± 0.2	0.5 ± 0.1	14.3 ± 0.9

^a^ Calculated with ChemDraw 11.0 software; ^b^ Liver carcinoma; ^c^ Bladder carcinoma; ^d^ Colon carcinoma; ^e^ Leukemia; ^f^ ND indicated that no appreciable inhibition value (IC_50_) was observed upon treatment of maximal concentration at 100 μM. Data are expressed as the mean ± SD from the dose response curve of at least three independent experiments; ^g^ -: No test.

In light of the cytotoxicity findings described above, we extended our study on the anti-proliferation efficacy of compounds **9** and **18** against HepG2 cells to 24 h, 48 h and 72 h periods. The data on norcantharidin (**2**) was used here as the base case. As shown in Table 2, first of all, the cytotoxicity of compounds **9** and **18** demonstrated, once again, an impressed fivefold and twofold, respectively, stronger bioactivity than that of norcantharidin (**2**) *in vitro*. In addition, the growth inhibition of HepG2 cells induced by these three samples was exhibited in a time-dependent manner.

**Table 2:** Cytotoxic effects of norcantharidin (**2**), compounds **9** and **18** against human HepG2 cancer cell lines for 24, 48, and 72 h.

Compound	IC_50_ (μM) ^a^ (Mean ± SD)		
	24 h	48 h	72 h
NCTD (**2**)	48.0 ± 0.7	42.0 ± 1.8	22.8 ± 0.6
**9**	10.7 ± 0.2 ***	8.3 ± 1.3 ***	8.2 ± 0.6 ***
**18**	19.4 ± 2.1 **	16.4 ± 1.2 ***	12.8 ± 2.5 ***

^a^ Data are expressed as the mean ± SD from the dose response curve of at least three independent experiments. ** *p* < 0.01, *** *p* < 0.001 *versus* NCTD (**2**).

### 2.3. Nuclear Morphological Changes of HepG2 Cells Treated with Norcantharidin (**2**), Compounds **9** and **18**

**Figure 2:**
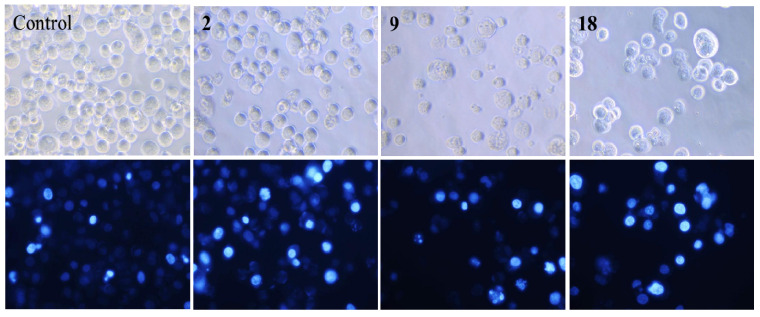
(Top) Cell morphological observations of nuclear change of human hepatoma HepG2 cell lines after 48 h exposure (untreated), with 40 μM norcantharidin (**2**), compounds **9** and **18**. (Bottom) Cells were stained with Hoechst 33258 and examined using a Nikon (Tokyo, Japan) fluorescence microscope. (Magnification, 400×).

To further investigate the role of apoptosis in the cytotoxicity of *N*-farnesyloxy- (**9**) and *N*-farnesyl-norcantharimide (**18**), we incubated HepG2 cells with norcantharidin (**2**), compounds **9** or **18**, separately, for 48 h. The cells were then stained with Hoechst 33,258, and examined by fluorescence microscopy for topical morphological changes [41]. As shown in Figure 2, while the nuclei of the cells were round in shape and stained homogenously in the control without testing compound, those treated with norcantharidin (**2**), *N*-farnesyloxy- (**9**) and *N*-farnesylnorcantharimide (**18**) showed typical morphological features of apoptosis such as cell shrinkage, chromatin condensation and DNA fragmentation [16]. Evidently, the proliferation of the cancer cell was inhibited by the testing compounds *in vitro*.

### 2.4. Cell Cycle Distribution Analysis Using Flow Cytometry

**Figure 3:**
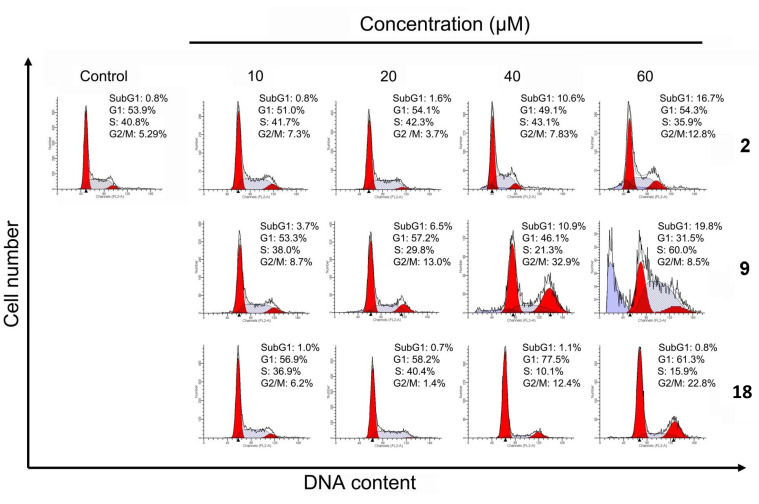
Induction of cells arrest by norcantharidin (**2**), compounds **9** and **18**. Effects of different concentrations of norcantharidin (**2**), compounds **9** and **18** on cell-cycle progression of HepG2 cells. HepG2 cells were untreated or treated with 10–60 μM norcantharidin (**2**), compounds **9** and **18** for 48 h. After treatment, cells were fixed and stained with PI, and the cell cycle distribution was examined by flow cytometer.

To probe the apoptotic effects of norcantharidin (**2**), compounds **9** and **18** on cell cycle progression, HepG2 cells were treated once again with these compounds, separately, at different concentrations for 48 h. The cell cycle distribution of the cancer cells was analyzed by flow cytometry, and the subG1 phase was analyzed by flow cytometry with propidium iodide (PI) staining [42]. The control of the experiment was the untreated cells. As illustrated in Figure 3, only a small fraction of apoptotic cells (0.8%) was detected in the control as well as in norcantharidin (**2**) at a low concentration of 10 μM. However, as norcantharidin (**2**) dosage was increased from 20 to 60 μM, the fraction of apoptotic cell went up substantially from 1.16% to 16.7% in a dose-dependent manner. As for compound **18**, the apoptotic effect meagerly laid between 0.7% to 1.1% across the treatment range of 10–60 μM. On the other hand, compound **9** gave rise to an impressive 3.7%–19.7% apoptosis from 10 to 60 μM and induced cell accumulation in the G2/M phase.

### 2.5. Apoptotic Analyses-Annexin V-FITC/PI Double Staining and Flow Cytometry Analyses

**Figure 4:**
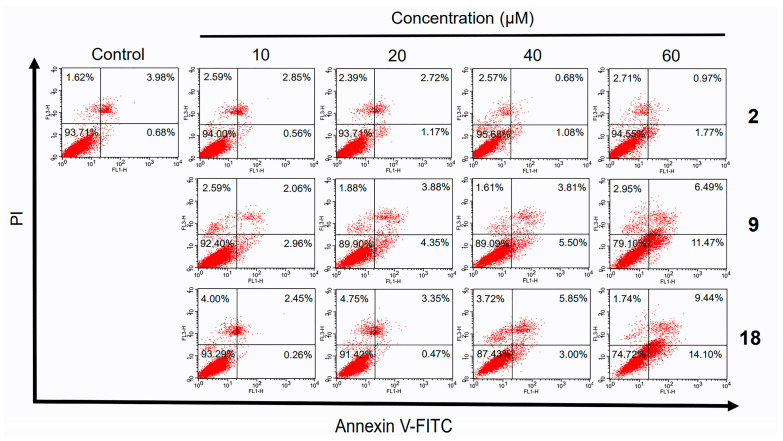
Flow cytometry analysis of HepG2 cells treated with different concentrations of norcantharidin (**2**), compounds **9** and **18** for 48 h. Treated cells were examined for apoptotic cells using Annexin V-FITC apoptosis detection kit. Annexin V-positive/PI-negative cells were in early stages of apoptosis and double-positive cells were in late stages of apoptosis, whereas annexin V-negative/PI-positive cells were necrotic.

To study in depth the bioactivities of norcantharidin (**2**), compounds **9** and **18** against HepG2 cells, the cancer cells were treated with vehicle alone as control or with one of the three testing compounds at different concentrations (10–60 μM). After 48 h, the samples were double-stained with annexin V-FITC and PI [42]. The percentages of cell populations at various stages of apoptosis were exhibited in Figure 4. Evidently the data pointed out that the distributions of apoptotic cell death resulting from the treatment of compounds **9** and **18** were dose-dependent, but this was not the case for norcantharidin (**2**). Starting from a dosage of 40 μM, both compounds **9** and **18** induced higher frequency of HepG2 cells apoptosis, as well as cytotoxic effects at both early and late stages by annexin V-FITC/PI staining analysis. For norcantharidin (**2**), still the only discernible effect was seen at a higher threshold (60 μM). We attributed this finding to and confirmed that the superior efficiency of both compounds **9** and **18** lies in its cytotoxicity and inhibitive function on human hepatoma cell proliferation.

## 3. Experimental

### 3.1. General

In details, all chemical reagents in commercial quality were used as received (Sigma-Aldrich or Acros Organics) and were used without further purification. Solvents were dried and the synthesized compounds were purified using standard techniques. In general, the reactions were carried out under anhydrous conditions in dry solvent and nitrogen atmosphere. Reactions-progression was monitored by thin layer chromatography (TLC) on aluminum plates coated with silica gel with a fluorescent indicator (Merck 60 F_254_). Unless otherwise stated, column chromatography was performed with silica gel Silia*Flash*^®^ G60 (60–200 μm) purchased from SiliCycle Inc. (Quebec City, QC, Canada). Melting points were determined in open capillaries using the Fargo MP-2D apparatus and were uncorrected. NMR spectra were recorded in CDCl_3_ at 500.13 MHz for ^1^H and at 125.77 MHz for ^13^C (Bruker AVANCE III plus 500 MHz), respectively. Chemical shift (*δ*) were reported in parts per million (ppm) measured relative to the internal standards (TMS), and the coupling constant (*J*) were expressed in Hertz (Hz). The purity of these compounds was more than 98% based on the analysis of HPLC with RP-C_18_ column. The mass spectra were acquired using a Thermo Finnigan model LXQ (Thermo Electron Co., Waltham, MA, USA) ion trap mass spectrometer equipped with ESI source interference and controlled by Xcalibur 2.06. The mass spectra were acquired in a positive ion mode or a negative ion mode.

### 3.2. General Procedure for the Preparation of Compounds **2**, **5** and **6**

*7-Oxabicyclo[2.2.1]-5-heptene-2,3-dicarboxylic anhydride* (**5**). A solution of furan (40 mL, 550 mmol) and maleic anhydride (10 g, 102 mmol) were stirred together in ether (100 mL) at room temperature for 48 h, after which the white precipitate was collected to give **5** as a colorless solid, yield 93.3%, mp 121–122 °C. ^1^H-NMR (CDCl_3_): *δ* 6.58 (s, 2H, H-5,6), 5.47 (s, 2H, H-1,4), 3.19 (s, 2H, H-2,3); ^13^C-NMR (CDCl_3_): *δ* 170.1, 137.2, 82.4, 48.9; LC-MS (ESI^‒^, *m/z*) calculated for C_8_H_6_O_4_: 166.03, found for 164.90 [M–H]^‒^.

*7-Oxabicyclo[2.2.1]heptane-2,3-dicarboxylic anhydride* (**2**). To a solution of **5** (4.7 g, 28.3 mmol) in THF (200 mL), 10% Pd/C (470 mg) was added, and the mixture was stirred at room temperature under a hydrogen atmosphere (3 atm) for 8–12 h. The reaction mixture was filtered through Celite 545^®^ and concentrated *in vacuo* to give norcantharidin (**2**) as colorless crystals, yield 91.4%, mp 116–118 °C. ^1^H-NMR (CDCl_3_): *δ* 5.05 (t, *J* = 2.3 Hz, 2H, H-1,4), 3.18 (s, 2H, H-2,3), 1.91–1.89 (m, 2H, H-5,6), 1.65–1.63 (m, 2H, H-5,6); ^13^C-NMR (CDCl_3_): *δ* 171.1, 80.2, 50.6, 28.1; LC-MS (ESI^‒^, *m/z*) calculated for C_8_H_8_O_4_: 168.04, found for 166.86 [M–H]^‒^.

*N-Hydroxy-7-oxabicyclo[2.2.1]heptane-2,3-dicarboximide* (**6**). A solution of **2** (5.04 g, 30 mmol) in dry methanol (200 mL), sodium methoxide (1.62 g, 30 mmol) and hydroxylamine hydrochloride (2.08 g, 30 mmol) was added, and the mixture was stirred at room temperature for 20 h. The reaction mixture was filtered and concentrated *in vacuo* and recrystallized using CHCl_3_ to give **6** as a colorless crystals, yield 63%, mp 168‒169 °C. ^1^H-NMR (D_2_O): *δ* 4.93 (s, 2H, H-1,4), 3.21 (s, 2H, H-2,3), 1.89–1.86 (m, 2H, H-5,6), 1.78–1.74 (m, 2H, H-5,6); ^13^C-NMR (D_2_O): *δ* 177.0, 78.8, 46.9, 28.0; LC-MS (ESI^+^, *m/z*) calculated for C_8_H_9_NO_4_: 183.05, found for 206.00 [M+Na]^+^.

### 3.3. General Procedure for Synthesis of Target Compounds **7**‒**12**

To a stirred solution of **5** or **6** (1 mmol) in dry acetone (25 mL) was added the appropriate alkyl or terpenyl bromide (1 mmol) and K_2_CO_3_ (3 mmol), and the reaction mixture was refluxed for 8‒10 h. Then, the reaction mixture was filtered and concentrated *in vacuo*, and the residue was purified by column chromatography using silica gel with ethyl acetate/*n*-hexane as eluent to afford the desired compounds.

*N-Isoprenyloxy-7-oxabicyclo[2.2.1]heptane-2,3-dicarboximide* (**7**). Colorless crystals, yield 64%, mp 93–94 °C. ^1^H-NMR (CDCl_3_): *δ* 5.38 (t, *J* = 7.7 Hz, 1H, H-2'), 4.83 (t, *J* = 2.7 Hz, 2H, H-1,4), 4.57 (d, *J* = 7.7 Hz, 2H, H-1'), 2.80 (s, 2H, H-2,3), 1.86–1.83 (m, 2H, H-5,6), 1.74 (s, 3H, CH_3_), 1.69 (s, 3H, CH_3_), 1.59–1.56 (m, 2H, H-5,6); ^13^C-NMR (CDCl_3_): *δ* 171.9, 144.3, 116.7, 78.8, 73.3 47.6, 28.9, 26.1, 18.2; LC-MS (ESI^+^, *m/z*) calculated for C_13_H_17_NO_4_: 251.12, found for 274.05 [M+Na]^+^.

*N-Geranyloxy-7-oxabicyclo[2.2.1]heptane-2,3-dicarboximide* (**8**). Colorless liquid, yield 56%. ^1^H-NMR (CDCl_3_): *δ* 5.40 (t, *J* = 7.6 Hz, 1H, H-2'), 5.05 (t, *J* = 6.0 Hz, 1H, H-6'), 4.86 (t, *J* = 2.6 Hz, 2H, H-1,4), 4.63 (d, *J* = 7.6 Hz, 2H, H-1'), 2.83 (s, 2H, H-2,3), 2.11–2.02 (m, 4H, H-5,6, H-4'), 1.90–1.86 (m, 2H, H-5'), 1.72 (s, 3H, CH_3_), 1.68 (s, 3H, CH_3_), 1.59 (s, 3H, CH_3_), 1.6–1.58 (m, 2H, H-5,6); ^13^C-NMR (CDCl_3_): *δ* 171.9, 147.5, 132.1, 123.9, 116.4, 78.8, 73.3, 47.6, 39.9, 29.0, 26.4, 25.9, 17.9, 16.8; LC-MS (ESI^+^, *m/z*) calculated for C_18_H_25_NO_4_: 319.18, found for 342.13 [M+Na]^+^.

*N-Farnesyloxy-7-oxabicyclo[2.2.1]heptane-2,3-dicarboximide* (**9**). Colorless liquid, yield 28%. ^1^H-NMR (CDCl_3_): *δ* 5.42 (td, *J* = 0.9, 7.7 Hz, 1H, H-2'), 5.11–5.07 (m, 2H, H-6',10'), 4.85 (q, *J* = 2.4 Hz, 2H, H-1,4), 4.62 (d, *J* = 7.7 Hz, 2H, H-1'), 2.83 (s, 2H, H-2,3), 2.09–2.04 (m, 6H, H-5,6, H-4',8'), 1.99–1.96 (m, 2H, H-5'), 1.89–1.86 (m, 2H, H-6'), 1.72 (d, *J* = 0.7 Hz, 3H, CH_3_), 1.68 (s, 3H, CH_3_), 1.63–1.62 (m, 2H, H-5,6), 1.60 (s, 3H, CH_3_), 1.59 (s, 3H, CH_3_); ^13^C-NMR (CDCl_3_): *δ* 171.6, 147.3, 135.5, 131.3, 124.3, 123.5, 116.1, 78.5, 73.0, 47.4, 39.65, 39.63, 28.7, 26.7, 26.1, 25.7, 17.7, 16.5, 16.0; LC-MS (ESI^+^, *m/z*) calculated for C_23_H_33_NO_4_: 387.24, found for 410.21 [M+Na]^+^.

*N-Butyloxy-7-oxabicyclo[2.2.1]heptane-2,3-dicarboximide* (**10**). Colorless solid, yield 24%, mp 113–114 °C. ^1^H-NMR (CDCl_3_): *δ* 4.86 (dd, *J* = 2.3, 2.4 Hz, 2H, H-1,4), 4.04 (t, *J* = 6.8 Hz, 2H, H-1'), 2.82 (s, 2H, H-2,3), 1.87–1.85 (m, 2H, H-5,6), 1.67 (p, *J* = 6.8 Hz, 2H, H-2'), 1.64–1.57 (m, 2H, H-5,6), 1.43 (hexa, *J* = 7.4 Hz, 2H, H-3'), 0.92 (t, *J* = 7.4 Hz, 3H, H-4'); ^13^C-NMR (CDCl_3_): *δ* 171.4, 78.7, 77.2, 47.3, 29.9, 28.7, 18.7, 13.7; LC-MS (ESI^+^, *m/z*) calculated for C_12_H_17_NO_4_: 239.12, found for 240.24 [M+H]^+^.

*N-Octyloxy-7-oxabicyclo[2.2.1]heptane-2,3-dicarboximide* (**11**). Colorless solid, yield 22%, mp 96–97 °C. ^1^H-NMR (CDCl_3_): *δ* 4.89 (dd, *J* = 2.3, 2.4 Hz, 2H, H-1,4), 4.05 (t, *J* = 6.9 Hz, 2H, H-1'), 2.85 (s, 2H, H-2,3), 1.91–1.87 (m, 2H, H-5,6), 1.69 (p, *J* = 6.9 Hz, 2H, H-2'), 1.64–1.60 (m, 2H, H-5,6), 1.43–1.38 (m, 2H, H-3'), 1.32–1.26 (m, 8H, H-4'-7'), 0.88 (t, *J* = 6.8 Hz, 3H, H-12'); ^13^C-NMR (CDCl_3_): *δ* 171.4, 78.7, 77.6, 47.3, 31.7, 29.2, 29.1, 28.7, 27.9, 25.4, 22.6, 14.1; LC-MS (ESI^+^, *m/z*) calculated for C_16_H_25_NO_4_: 295.18, found for 296.24 [M+H]^+^.

*N-Dodecyloxy-7-oxabicyclo[2.2.1]heptane-2,3-dicarboximide* (**12**)*.* Colorless solid, yield 40%, mp 90–91 °C. ^1^H-NMR (CDCl_3_): *δ* 4.89 (dd, *J* = 2.3, 2.4 Hz, 2H, H-1,4), 4.05 (t, *J* = 6.9 Hz, 2H, H-1'), 2.85 (s, 2H, H-2,3), 1.91–1.87 (m, 2H, H-5,6), 1.73–1.67 (m, 2H, H-2'), 1.64–1.60 (m, 2H, H-5,6), 1.44–1.38 (m, 2H, H-3'), 1.30–1.26 (m, 16H, H-4'-11'), 0.88 (t, *J* = 6.8 Hz, 3H, H-12'); ^13^C-NMR (CDCl_3_): *δ* 171.4, 78.7, 77.6, 47.3, 31.9, 29.6, 29.5, 29.4, 29.32, 29.26, 28.7, 27.9, 25.4, 22.7, 14.1; LC-MS (ESI^+^, *m/z*) calculated for C_20_H_33_NO_4_: 351.24, found for 374.26 [M+Na]^+^. 

*7-Oxabicyclo[2.2.1]-5-heptene-2,3-dicarboximide* (**14**). A solution of maleimide (**13**) (1.5 g, 15 mmol) in dry toluene (30 mL) was heated at 80 °C, then furan (5.4 mL, 75 mmol) was added and stirred at 80 °C for 6 h, cooled at room temperature and white precipitated was collected and purified by column chromatography using silica gel (1:3 ethyl acetate/*n*-hexane as eluent) to give **14** (2.1 g, yield: 87%) as a colorless crystals, mp 162–163 °C. ^1^H-NMR (CDCl_3_): *δ* 8.13 (s, 1H, NH), 6.50 (s, 2H, H-5,6), 5.29 (s, 2H, H-1,4), 2.87 (s, 2H, H-2,3); ^13^C-NMR (CDCl_3_): *δ* 176.2, 136.8, 81.2, 48.9; LC-MS (ESI^‒^, *m/z*) calculated for C_8_H_7_NO_3_: 165.04, found for 164.02 [M–H]^‒^.

*7-Oxabicyclo[2.2.1]heptane-2,3-dicarboximide* (**15**). To a solution of **14** (0.5 g, 2.8 mmol) in THF (20 mL) was added 10% Pd/C (50 mg), and the mixture was stirred at room temperature under a hydrogen atmosphere for 4 h. The reaction mixture was filtered through Celite 545^®^ and concentrated *in vacuo* to give **15** (424 mg, yield: 84%) as a colorless crystals, mp 188–190 °C. ^1^H-NMR (CDCl_3_): *δ* 8.60 (s, 1H, NH), 4.91 (dd, *J* = 2.4, 3.3 Hz, 2H, H-1,4), 2.92 (s, 2H, H-2,3), 1.89–1.85 (m, 2H, H-5,6), 1.61–1.57 (m, 2H, H-5,6); ^13^C-NMR (CDCl_3_): *δ* 177.2, 79.1, 51.3, 28.5; LC-MS (ESI^‒^, *m/z*) calculated for C_8_H_9_NO_3_: 167.06, found for 166.03 [M–H]^‒^.

### 3.4. General Procedure for Synthesis of Target Compounds **16**–**24**

A mixture of **14** or **15** (1.0 mmol) in dry acetone (25 mL) was added the appropriate alkyl or terpenyl bromide (1.0 mmol) and K_2_CO_3_ (3 mmol), and the reaction mixture was refluxed for 8–10 h. Then, the reaction mixture was filtered and concentrated *in vacuo*, and the residue was purified by column chromatography using silica gel with ethyl acetate/*n*-hexane as eluent to afford the desired compounds.

*N-Isoprenyl-7-oxabicyclo[2.2.1]heptane-2,3-dicarboximide* (**16**). White solid, yield 36%, mp 87–88 °C. ^1^H-NMR (CDCl_3_): *δ* 5.09 (t, *J* = 7.1 Hz, 1H, H-2'), 4.85 (dd, *J* = 2.3, 3.0 Hz, 2H, H-1,4), 4.02 (d, *J* = 7.1 Hz, 2H, H-1'), 2.83 (s, 2H, H-2,3), 1.85–1.81 (m, 2H, H-5,6), 1.72 (s, 3H, CH_3_), 1.67 (s, 3H, CH_3_), 1.59–1.55 (m, 2H, H-5,6); ^13^C-NMR (CDCl_3_): *δ* 176.9, 137.4, 117.4, 79.0, 50.0, 37.0, 28.6, 25.6, 17.9; LC-MS (ESI^+^, *m/z*) calculated for C_13_H_17_NO_3_: 235.12, found for 235.12 [M+Na]^+^.

*N-Geranyl-7-oxabicyclo[2.2.1]heptane-2,3-dicarboximide* (**17**). White solid, yield 24%, mp 58–59 °C. ^1^H-NMR (CDCl_3_): *δ* 5.09 (t, *J* = 7.0 Hz, 1H, H-2'), 5.03 (t, *J* = 6.8 Hz, 1H, H-6'), 4.84 (t, *J* = 2.3 Hz, 2H, H-1,4), 4.03 (d, *J* = 7.0 Hz, 2H, H-1'), 2.83 (s, 2H, H-2,3), 2.05–2.00 (m, 2H, H-5'), 1.97–1.94 (m, 2H, H-4'), 1.84–1.82 (m, 2H, H-5,6), 1.72 (s, 3H, CH_3_), 1.64 (s, 3H, CH_3_), 1.59–1.55 (m, 2H, H-5,6), 1.56 (s, 3H, CH_3_); ^13^C-NMR (CDCl_3_): *δ* 177.0, 141.1, 131.9, 124.0, 117.5, 79.2, 50.2, 39.7, 37.2, 28.8, 26.5, 25.9, 17.9, 16.6; LC-MS (ESI^+^, *m/z*) calculated for C_18_H_25_NO_3_: 303.18, found for 326.15 [M+Na]^+^.

*N-Farnesyl-7-oxabicyclo[2.2.1]heptane-2,3-dicarboximide* (**18**). Colorless liquid, yield 20%. ^1^H-NMR (CDCl_3_): *δ* 5.10–5.02 (m, 3H, H-2',6',10'), 4.84 (dd, *J* = 2.2, 2.3 Hz, 2H, H-1,4), 4.03 (d, *J* = 7.0 Hz, 2H, H-1'), 2.82 (s, 2H, H-2,3), 2.06–2.01 (m, 4H, H-4',8'), 1.97–1.92 (m, 4H, H-5',9'), 1.84–1.81 (m, 2H, H-5,6), 1.75 (s, 3H, CH_3_), 1.67 (s, 3H, CH_3_), 1.60 (s, 3H, CH_3_), 1.59 (s, 3H, CH_3_), 1.58–1.55 (m, 2H, H-5,6); ^13^C-NMR (CDCl_3_): *δ* 176.8, 140.9, 135.3, 131.2, 124.3, 123.7, 117.2, 78.9, 50.0, 39.7, 39.5, 37.0, 28.6, 26.8, 26.3, 25.7, 17.7, 16.4, 16.0; LC-MS (ESI^+^, *m/z*) calculated for C_23_H_33_NO_3_: 371.25, found for 394.20 [M+Na]^+^.

*N-Butyl-7-oxabicyclo[2.2.1]heptane-2,3-dicarboximide* (**19**). Colorless liquid, yield 67%. ^1^H-NMR (CDCl_3_): *δ* 4.82 (t, *J* = 2.4 Hz, 2H, H-1,4), 3.42 (t, *J* = 7.4 Hz, 2H, H-1'), 2.81 (s, 2H, H-2,3), 1.83–1.80 (m, 2H, H-5,6), 1.58–1.54 (m, 2H, H-5,6), 1.51–1.45 (m, 2H, H-2'), 1.25 (sep, *J* = 7.4 Hz, 2H, H-3'), 0.87 (t, *J* = 7.4 Hz, 3H, H-4'); ^13^C-NMR (CDCl_3_): *δ* 177.3, 79.0, 49.9, 38.6, 29.6, 28.6, 19.9, 13.6; LC-MS (ESI^+^, *m/z*) calculated for C_12_H_17_NO_3_: 223.12, found for 246.15 [M+Na]^+^.

*N-Octyl-7-oxabicyclo[2.2.1]heptane-2,3-dicarboximide* (**20**). Colorless liquid, yield 52%. ^1^H-NMR (CDCl_3_): *δ* 4.87 (dd, *J* = 2.3, 2.4 Hz, 2H, H-1,4), 3.45 (t, *J* = 7.5 Hz, 2H, H-1'), 2.85 (s, 2H, H-2,3), 1.87–1.84 (m, 2H, H-5,6), 1.62–1.60 (m, 2H, H-5,6), 1.55–1.52 (m, 2H, H-2'), 1.27–1.25 (m, 10H, H-3'–7'), 0.87 (t, *J* = 7.0 Hz, 3H, H-8'); ^13^C-NMR (CDCl_3_): *δ* 177.2, 79.0, 49.9, 39.1, 31.7, 29.1, 29.0, 28.6, 27.6, 26.7, 22.6, 14.1; LC-MS (ESI^+^, *m/z*) calculated for C_18_H_25_NO_3_: 303.18, found for 302.20 [M‒H]^+^.

*N-Dodecyl-7-oxabicyclo[2.2.1]heptane-2,3-dicarboximide* (**21**). Colorless liquid, yield 77%. ^1^H-NMR (CDCl_3_): *δ* 4.86 (dd, *J* = 2.3, 3.0 Hz, 2H, H-1,4), 3.45 (t, *J* = 7.5 Hz, 2H, H-1'), 2.85 (s, 2H, H-2,3), 1.87–1.83 (m, 2H, H-5,6), 1.62–1.58 (m, 2H, H-5,6), 1.53 (p, *J* = 6.8 Hz, 2H, H-2'), 1.24 (m, 18H, H-3'–11'), 0.88 (t, *J* = 6.9 Hz, 3H, H-12'); ^13^C-NMR (CDCl_3_): *δ* 177.2, 79.0, 49.8, 39.1, 31.7, 29.6, 29.5, 29.4, 29.3, 29.1, 28.6, 27.5, 26.6, 22.6, 14.1; LC-MS (ESI^+^, *m/z*) calculated for C_20_H_33_NO_3_: 335.25, found for 358.36 [M+Na]^+^.

*N-Isoprenyl-7-oxabicyclo[2.2.1]-5-heptene-2,3-dicarboximide* (**22**) White solid, yield 93%, mp 111–112 °C. ^1^H-NMR (CDCl_3_): *δ* 6.48 (s, 2H, H-5,6), 5.24 (s, 2H, H-1,4), 5.10 (td, *J* = 1.1, 7.0 Hz, 1H, H-2'), 4.05 (d, *J* = 7.0 Hz, 2H, H-1'), 2.80 (s, 2H, H-2,3), 1.73 (s, 3H, CH_3_), 1.67 (s, 3H, CH_3_); ^13^C-NMR (CDCl_3_): *δ* 176.1, 137.6, 136.8, 117.6, 81.1, 47.7, 37.2, 25.8, 18.2; LC-MS (ESI^+^, *m/z*) calculated for C_13_H_15_NO_3_: 233.11, found for 256.06 [M+Na]^+^.

*N-Geranyl-7-oxabicyclo[2.2.1]-5-heptene-2,3-dicarboximide* (**23**) White solid, yield 53%, mp 80–81 °C. ^1^H-NMR (CDCl_3_): *δ* 6.48 (s, 2H, H-5,6), 5.24 (s, 2H, H-1,4), 5.10 (t, *J* = 6.9 Hz, 1H, H-2'), 5.02 (t, *J* = 6.8 Hz, 1H, H-6'), 4.04 (d, *J* = 6.9 Hz, 2H, H-1'), 2.80 (s, 2H, H-2,3), 2.04–2.00 (m, 2H, H-4'), 1.97–1.94 (m, 2H, H-5'), 1.75 (s, 3H, CH_3_), 1.66 (s, 3H, CH_3_), 1.58 (s, 3H, CH_3_); ^13^C-NMR (CDCl_3_): *δ* 176.1, 141.1, 136.8, 131.9, 124.0, 117.4, 81.1, 47.7, 39.7, 37.1, 26.5, 25.9, 17.9, 16.6; LC-MS (ESI^+^, *m/z*) calculated for C_18_H_23_NO_3_: 301.17, found for 324.05 [M+Na]^+^.

*N-Farnesyl-7-oxabicyclo[2.2.1]-5-heptene-2,3-dicarboximide* (**24**) White solid, yield 30%, mp 76–77 °C. ^1^H-NMR (CDCl_3_): *δ* 6.48 (s, 2H, H-5,6), 5.24 (s, 2H, H-1,4), 5.13 (dt, *J* = 0.8, 7.0 Hz, 1H, H-2'), 5.07–5.02 (m, 2H, H-6',10'), 4.04 (d, *J* = 7.0 Hz, 2H, H-1'), 2.80 (s, 2H, H-2,3), 2.04–2.01 (m, 4H, H-4',8'), 2.00–1.91 (m, 4H, H-5',9'), 1.73 (s, 3H, CH_3_), 1.65 (s, 3H, CH_3_), 1.57 (s, 3H, CH_3_), 1.55 (s, 3H, CH_3_); ^13^C-NMR (CDCl_3_): *δ* 176.1, 141.1, 136.8, 135.5, 131.5, 124.6, 123.9, 117.4, 81.1, 47.7, 39.9, 39.7, 37.1, 26.9, 26.5, 25.9, 17.9, 16.6, 16.2; LC-MS (ESI^+^, *m/z*) calculated for C_23_H_31_NO_3_: 369.23, found for 392.08 [M+Na]^+^.

### 3.5. General Procedure for Synthesis of Target Compounds **25**–**27**

A solution of **22**–**24** (0.86 mmol) in THF (15 mL) was added 10% Pd/C (10 mg), and the mixture was stirred at room temperature under a hydrogen atmosphere for 48 h. The reaction mixture was filtered through Celite 545^®^ and concentrated *in vacuo* and purified by column chromatography using silica gel with ethyl acetate/*n*-hexane as eluent to afford the desired compounds.

*N-3′-Methylbutyl-7-oxabicyclo[2.2.1]heptane-2,3-dicarboximide* (**25**). White solid, yield 86%, mp 52–53 °C. ^1^H-NMR (CDCl_3_): *δ* 4.87 (dd, *J* = 2.3, 3.1 Hz, 2H, H-1,4), 3.47 (t, *J* = 7.7 Hz, 2H, H-1'), 2.85 (s, 2H, H-2,3), 1.84–1.81 (m, 2H, H-5,6), 1.61–1.56 (m, 2H, H-5,6), 1.54 (nonet, *J* = 6.6 Hz, 1H, H-3'), 1.42 (dd, *J* = 6.6, 7.7 Hz, 2H, H-2'), 0.92 (d, *J* = 6.6 Hz, 6H, 2 × CH_3_); ^13^C-NMR (CDCl_3_): *δ* 177.4, 79.2, 50.1, 37.8, 36.5, 28.8, 26.1, 22.5; LC-MS (ESI^+^, *m/z*) calculated for C_13_H_19_NO_3_: 237.14, found for 260.17 [M+Na]^+^.

*N-3′,7′-Dimethyloctyl-7-oxabicyclo[2.2.1]heptane-2,3-dicarboximide* (**26**). Colorless liquid, yield 78%. ^1^H-NMR (CDCl_3_): *δ* 4.87 (dd, *J* = 2.2, 3.2 Hz, 2H, H-1,4),3.47 (td, *J* = 2.1, 6.2 Hz, 2H, H-1'), 2.85 (s, 2H, H-2,3), 1.86–18.4 (m, 2H, H-5,6), 1.61–1.56 (m, 2H, H-5,6), 1.56–1.54 (m, 1H, H-3'), 1.53–1.46 (m, 1H, H-7'), 1.42–1.07 (m, 8H, H-2',4',5',6'), 0.90 (d, *J* = 6.5 Hz, 3H, CH_3_), 0.86 (dd, *J* = 6.6 Hz, 6H, 2 × CH_3_); ^13^C-NMR (CDCl_3_): *δ* 177.4, 79.2, 50.1, 39.4, 37.6, 37.1, 34.7, 30.9, 28.8, 28.1, 24.7, 22.9, 22.8, 19.5; LC-MS (ESI^+^, *m/z*) calculated for C_18_H_29_NO_3_: 307.21, found for 330.19 [M+Na]^+^.

*N-3′,7′,11′-Trimethyldodecyl-7-oxabicyclo[2.2.1]heptane-2,3-dicarboximide* (**27**). Colorless liquid, yield 25%. ^1^H-NMR (CDCl_3_): *δ* 4.87 (dd, *J* = 2.2, 3.0 Hz, 2H, H-1,4), 3.49–3.45 (m, 2H, H-1'), 2.85 (s, 2H, H-2,3), 1.86–1.84 (m, 2H, H-5,6), 1.61–1.56 (m, 2H, H-5,6), 1.56–1.47 (m, 3H, H-3',7',11'), 1.40–1.00 (m, 14H, H-2',4',5',6',8',9',10'), 0.91 (d, *J* = 6.5 Hz, 3H, CH_3_), 0.86 (d, *J* = 6.6 Hz, 6H, 2 × CH_3_), 0.83 (dd, *J* = 0.9, 6.6 Hz, 3H, CH_3_); ^13^C-NMR (CDCl_3_): *δ* 177.2, 79.0, 49.9, 39.5, 37.4, 37.4, 37.3, 37.0, 34.5, 32.7, 30.7, 28.6, 28.0, 24.8, 24.2, 22.7, 22.6, 19.7, 19.3; LC-MS (ESI^+^, *m/z*) calculated for C_23_H_39_NO_3_: 377.29, found for 400.28 [M+Na]^+^.

### 3.6. In Vitro Pharmacology

#### 3.6.1. Cell Culture and Stock Solutions

Stock solutions were prepared as follows and stored at −20 °C: norcantharidin (2) as a 30 mM solution in dimethylsulfoxide (DMSO) and norcantharimide derivatives as 20 mM solution in DMSO. All cell lines were maintained in Dulbecco’s modified Eagle’s medium (DMEM, HyClone, Logan, UT, USA) and RPMI 1640 (HyClone, USA), supplemented with 10% heat-inactivated fetal bovine serum (FBS) and 1% penicillin/streptomycin in CO_2_ incubator with a humidified atmosphere of 95% air and 5% CO_2_ at 37 °C.

#### 3.6.2. Cell Cytotoxicity Assay Using MTT Assay

The cytotoxic activities of compounds were evaluated using HepG2 (liver carcinoma), BFTC905 (bladder carcinoma), HT-29 (colon carcinoma), SW480 (colon carcinoma), and HL-60 (leukemia). The cytotoxic activities were assessed by the MTT (3-[4,5-dimethylthiazol-2-yl]-2,5-diphenyltetrazolium bromide) assay [41]. HepG2, BFTC905, HT-29, SW480, and HL-60 cell lines were cultured at 37 °C under 5% CO_2_ in air and were maintained in DMEM medium or RPMI 1640 medium, supplemented with 10% FBS, 10 mM sodium bicarbonate, penicillin (100 IU/mL), streptomycin (100 mg/mL) and glutamine (4 mM). The compounds were dissolved in DMSO as 20 mM stock solutions and diluted with culture medium used before. The final concentration of DMSO in the medium was less than 0.2% and it showed no interference with the biological activities tested. After the cells were seeded for 24 h, the compounds were added and incubated for 48 h. Then 10 μL MTT (5 mg/mL, dissolved in culture medium) was added to each cell and incubated for 1 h (37 °C). The inhibition rate was calculated. The errors were quoted as standard deviations and three replicates were used in the calculation of these errors. Briefly, 2 × 10^4^ cells/well were seeded in 96-well plate; samples were then added to the wells at different concentrations keeping untreated and vehicle treated wells as controls. After 48 h incubation, 10 μL of MTT reagent (5 mg/mL) was added to the wells to facilitate reaction. Formazan crystals produced during the reaction were solubilized and the absorbance at 540 nm was measured with a microplate reader (Spectramax 340PC384, Molecular Devices, Orleans, CA, USA). The IC_50_ values were defined as the drug concentration that inhibits 50% cell growth by setting the viability of untreated cell as 100%. Values were represented as the mean ± SD from at least three independent experiments.

#### 3.6.3. Morphological Observations of Nuclear Change with Hoechst 33,258 Staining

Staining with Hoechst 33258 was performed according to the method described previously [27,41]. HepG2 cells were treated with 40 μM norcantharidin (**2**), compounds **9** or **18** for 48 h. The cells were washed with phosphate-buffered saline (PBS) and stained with Hoechst 33258 (Sigma, St. Louis, MO, USA) at a final concentration of 10 μg/mL. The slides were examined under a fluorescence microscope. Cells with a small nucleus, a high fluorescence intensity (due to chromatin condensation), or nuclear fragmentation were considered apoptotic.

#### 3.6.4. Flow Cytometry Analysis

Flow cytometry was used to obtain the cell cycle distribution and the apoptotic rate.To determine the effect of norcantharidin (**2**), compounds **9** or **18** on the cell cycle, parasites (1 × 10^6^ cells) were treated with norcantharidin (**2**), compounds **9** and **18** (10–60 μM) for 48 h. The cells were fixed in chilled 70% ethanol and kept at −20 °C until analysis. After the cells were washed in PBS, the resultant pellet was resuspended in 500 μL DNase (200 μg/mL) and incubated for 1.5 h at 37 °C. The cells were then stained with PI (40 μg/mL) and incubated in the dark for 20 min at 20–25 °C. Data acquisition was carried out using FACS scan and analyzed using the CellQuest pro software. 

The percentage of apoptotic cells was determined according to the manufacture’s protocol by using an annexin-V/FITC kit/propidium iodide (PI) flow cytometer [27,42]. To facilitate the detection of apoptosis, the treated cells were centrifuged for 5 min, under 1000 *g*, at room temperature (18–24 °C), and then resuspended and washed once with 5 mL phosphate-buffered saline before being stained with annexin-V/PI (apoptosis detection kit; R&D Systems, Taipei, Taiwan).

## 4. Conclusions

In summary, the lipophilicity of *N*-substituted norcantharimide derivatives plays a crucial role in their bioactivity. Thus, through optimization of the type and chain length of the *N*-substituent group we might be able to maximize the cytotoxicity of norcantharimide. We also found that compounds **9**, *N*-farnesyloxy-7-oxabicyclo[2.2.1]heptane-2,3-dicarboximide, and **18**, *N*-farnesyl-7-oxabicyclo[2.2.1]- heptane-2,3-dicarboximide, in our study had the highest cytotoxicity, anti-proliferative and apoptotic effects, among the twenty three samples studied, against HepG2 human hepatoma cell lines, without cytotoxic effect on murine embryonic liver BNL CL.2 cells. Should the same performance trends established here reproduced in clinic testing, compounds **9** and/or **18** could be promising candidates for anticancer drug development.

## Supplementary Files

Supplementary materials can be accessed at: http://www.mdpi.com/1420-3049/19/6/6911/s1.
