# Correction: Wu et al. Synthesis of Novel Lipophilic *N*-Substituted Norcantharimide Derivatives and Evaluation of Their Anticancer Activities. *Molecules* 2014, *19*, 6911–6928

**DOI:** 10.3390/molecules30112256

**Published:** 2025-05-22

**Authors:** Jin-Yi Wu, Cheng-Deng Kuo, Chien-Yu Chu, Min-Shin Chen, Jia-Hua Lin, Yu-Jen Chen, Hui-Fen Liao

**Affiliations:** 1 Department of Microbiology, Immunology and Biopharmaceutics, College of Life Sciences, National Chiayi University, Chiayi 60004, Taiwan; 2 Laboratory of Biophysics, Department of Medical Research, Taipei Veterans General Hospital, Taipei 11217, Taiwan; 3 Department of Radiation Oncology, Mackay Memorial Hospital, New Taipei City 25160, Taiwan; 4 Institute of Transitional Medicine, National Yang Ming University, Taipei 11221, Taiwan; 5 Department of Biochemical Science and Technology, College of Life Sciences, National Chiayi University, Chiayi 60004, Taiwan

## Error in Figure

In the original publication [1], there was a mistake in Figure 4 as published. The first picture in the second row and the second picture in the third row are mistyped. The corrected Figure 4 appears below.

The authors state that the scientific conclusions are unaffected. This correction was approved by the Academic Editor. The original publication has also been updated.

## Figures and Tables

**Figure 4:**
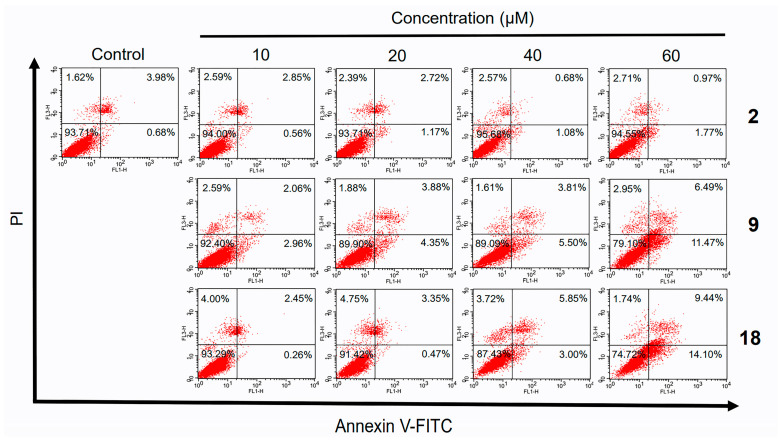
Flow cytometry analysis of HepG2 cells treated with different concentrations of norcantharidin (**2**), compounds **9** and **18** for 48 h. Treated cells were examined for apoptotic cells using Annexin V-FITC apoptosis detection kit. Annexin V-positive/PI-negative cells were in early stages of apoptosis and double-positive cells were in late stages of apoptosis, whereas annexin V-negative/PI-positive cells were necrotic.
